# Questioning the Role of Forward Associative Strength in False Memories: Evidence From Deese-Roediger-McDermott Lists With Three Critical Lures

**DOI:** 10.3389/fpsyg.2021.724594

**Published:** 2021-09-13

**Authors:** Maria Soledad Beato, Jason Arndt

**Affiliations:** ^1^Faculty of Psychology, University of Salamanca, Salamanca, Spain; ^2^Department of Psychology, Middlebury College, Middlebury, VT, United States

**Keywords:** false memory, Deese-Roediger-McDermott paradigm, multiple critical lures, forward associative strength, backward associative strength

## Abstract

We report an experiment examining the factors that produce false recognition in the Deese-Roediger-McDermott (DRM) paradigm. We selectively manipulated the probability that critical lures produce study items in free association, known as forward associative strength (FAS), while controlling the probability that study items produce critical lures in free association, known as backward associative strength (BAS). Results showed that false recognition of critical lures failed to differ between strong and weak FAS conditions. Follow-up correlational analyses further supported this outcome, showing that FAS was not correlated with false recognition, despite substantial variability in both variables across our stimulus sets. However, these correlational analyses did produce a significant and strong relationship between BAS and false recognition. These results support views that propose false memory is produced by activation spreading from study items to critical lures during encoding, which leads critical lures to be confused with episodically-experienced events.

## Introduction

False memory has been studied extensively using the Deese/Roediger-McDermott paradigm (DRM; Deese, 1959; Roediger and McDermott, 1995). In this paradigm, people study a list of words, which are all related to the same non-studied word known as the critical lure. On a subsequent memory test, critical lures are often mistakenly recalled or recognized (e.g., Arndt and Beato, 2017; Pitarque et al., 2018; Huff et al., 2020; Beato and Arndt, 2021). While this paradigm produces robust false memory, there is substantial variability in the false recognition that occurs across DRM lists (e.g., Gallo and Roediger, 2002; Beato and Díez, 2011; Cadavid and Beato, 2017; Coane et al., 2021).

In order to understand this variability, many studies have focused on the roles played by the probability that list items produce the critical lure in free association (referred to as backward associative strength or BAS) and the probability that the critical lure produces list items in free association (referred to as forward associative strength or FAS). This work has shown that BAS influences false memory reliably (e.g., McEvoy et al., 1999; Roediger et al., 2001; Gallo and Roediger, 2002; Arndt, 2006; Arndt and Gould, 2006; Howe et al., 2009a; Knott et al., 2012). On the contrary, prior work examining the effect of FAS on false memory has produced inconsistent results. Some studies did not find significant correlations between FAS and false recall/recognition (e.g., Roediger et al., 2001; Gallo and Roediger, 2002; Beato and Arndt, 2014), while other studies found correlations between FAS and false memory (e.g., Brainerd and Wright, 2005; Howe et al., 2009b; Arndt, 2012b, 2015). Brainerd and Wright (2005) argued that the lack of correlation found between FAS and false memory by, for example, Roediger et al. (2001) was due to the restricted range of FAS values used in their lists. Indeed, this same criticism applies to most studies showing that FAS fails to predict false memory (see Beato and Arndt, 2014 for an exception).

Beyond the empirical considerations highlighted above, the question of whether FAS impacts false memory is important theoretically. One class of theories, associative activation theories (Roediger et al., 2001; Howe et al., 2009b), posit that false memory in the DRM paradigm is caused by the activation spreading from study items’ representations in semantic memory to critical lures’ representations. As a result, these theories propose that BAS, but not FAS, should impact false memory. In contrast, other theories suggest that featural similarity between study items and critical lures (Arndt and Hirshman, 1998; Brainerd et al., 2008; Arndt, 2012a; Brainerd et al., 2020) increases false memory. In the view of these theories, both BAS and FAS should impact false memory, because both variables can be interpreted to index the extent to which study items and critical lures share features. Thus, investigating whether FAS influences false memory will help to distinguish between theoretical views of false memory’s genesis.

In the present study, we sought to determine whether FAS was related to false memory using lists that had substantial variability in FAS. In order to understand the unique role that FAS plays in producing false memories, it is important to separate the contributions of FAS and BAS to false memory, given that they are correlated (Brainerd et al., 2008). For this purpose, we built DRM lists that varied widely in FAS, while controlling BAS and employed DRM lists that were related to multiple critical lures (e.g., Beato et al., 2012; Cadavid et al., 2012; Beato and Arndt, 2014). Constructing lists that were related to multiple critical lures allowed us to evaluate correlations between FAS and false recognition using both the variability due to list-wide characteristics (the approach used in prior work, where lists are related to a single critical lure) and the variability due to individual critical lures’ characteristics as the basis for analyses. To illustrate, consider the lists that were related to the general theme “School,” we constructed one list of associates (homework, work, school, book, class, and test) that had strong FAS with three critical lures (assignment, lesson, and study), and a second list of associates (school, book, work, boring, long, and test) that had weak FAS with three critical lures (essay, homework, and study).^1^ Within a list that had a strong FAS-based relationship with its critical lures, there was variation in the summed FAS values of the three critical lures (e.g., assignment=0.755, lesson=0.294, and study=0.341). Importantly, lists that had a weak FAS-based relationship with its critical lures also varied in the summed FAS values of the three critical lures (e.g., essay=0.158, homework=0.265, and study=0.321).

The benefit of constructing lists this way is that it enabled us to examine the effects of study item characteristics and critical lure characteristics on false recognition separately. In contrast, the standard DRM paradigm, where each list of words is related to a single critical lure, only allows evaluation of these characteristics simultaneously, making it impossible to understand their unique impact on false memory. Thus, if the results of correlational analyses using study item and critical lure characteristics converge, it is appropriate to infer the variables correlated with false recognition reflect a general property of DRM lists, as well as the factors that drive false memory. On the other hand, if the results of correlational analyses produce different results using study item and critical lure characteristics as the unit of analysis, one may have less confidence that they reflect a general property of the factors that drive false memory, and instead may reflect specific item characteristics. As a consequence, factors that show the same effects for both sets of analyses are more likely to be key drivers of false memory effects, making them carry greater theoretical importance.

## Materials and Methods

### Participants

The sample comprised 40 native English speakers (70% female) who participated as part of a course research appreciation requirement. Participants’ ages ranged from 18 to 20years (*M age*=18.60; *SD*=0.78).

### Materials

A total of 28 six-word DRM lists were constructed as stimuli (see Table 1). Lists were built to ensure that three critical lures produced the same six study items in free association (i.e., they were related *via* FAS) based upon the University of South Florida free-association norms (Nelson et al., 1998). This list length was chosen because it allowed us the best opportunity to construct lists with multiple critical lures that manipulate FAS while controlling BAS across levels of FAS. While there is a tendency for critical lure false alarms to be lower with shorter lists, studying six associates of critical lures produces robust false memory (e.g., Robinson and Roediger, 1997).

**Table 1:** The 28 six-word study lists with three critical lures, the general theme for each pair of high- and low-FAS list, and the forward associative strength (FAS) condition for each list.

List	CRITICAL LURES: Associated words	General theme	FAS condition
List 1	ADVIL, EXCEDRIN, TYLENOL: headache, aspirin, medicine, pain, pill, drug	Medicine	High
List 2	OPERATION, SURGERY, TRAUMA: pain, hospital, blood, heart, hurt, sick	Medicine	Low
List 3	BOURBON, BRANDY, VODKA: drink, alcohol, liquor, drunk, whiskey, rum	Alcohol	High
List 4	BOTTLE, DRINK, WHISKEY: beer, drunk, liquor, wine, glass, alcohol	Alcohol	Low
List 5	COLONEL, CORPORAL, LIEUTENANT: army, sergeant, military, officer, captain, general	Army	High
List 6	COLONEL, COMMANDER, CORPORAL: sergeant, lieutenant, military, general, captain, officer	Army	Low
List 7	SOCCER, SOFTBALL, VOLLEYBALL: ball, game, sport, football, team, play	Sports	High
List 8	PLAYER, SPORTS, VOLLEYBALL: football, game, basketball, team, tennis, soccer	Sports	Low
List 9	BURIAL, BURY, GRAVE: dead, death, funeral, cemetery, die, ground	Death	High
List 10	DEAD, DEATH, DIE: sad, end, funeral, bury, heaven, grave	Death	Low
List 11	SHOWER, SPONGE, WASHCLOTH: clean, water, bath, soap, wet, wash	Soap	High
List 12	SPONGE, TOWEL, WASHCLOTH: water, bath, soap, wet, rag, wash	Soap	Low
List 13	CROPS, FARMER, HARVEST: corn, farm, food, wheat, field, vegetables	Food	High
List 14	CAKE, DESSERT, PASTRY: sweet, food, pie, fat, good, yummy	Food	Low
List 15	PURCHASE, SALE, SHOP: buy, store, clothes, money, mall, sell	Buy	High
List 16	PRODUCT, PURCHASE, SALE: buy, item, store, sell, car, price	Buy	Low
List 17	BURGLAR, THEFT, THIEF: steal, robber, crook, crime, criminal, bad	Crime	High
List 18	BURGLAR, FRAUD, ROBBERY: thief, steal, crime, money, crook, criminal	Crime	Low
List 19	JUPITER, NEPTUNE, URANUS: planet, mars, pluto, saturn, venus, space	Planet	High
List 20	JUPITER, NEPTUNE, PLUTO: mars, saturn, venus, uranus, moon, space	Planet	Low
List 21	ASSIGNMENT, LESSON, STUDY: homework, work, school, book, class, test	School	High
List 22	ESSAY, HOMEWORK, STUDY: school, book, work, boring, long, test	School	Low
List 23	NORM, NORMAL, ROUTINE: abnormal, average, same, regular, usual, boring	Normal	High
List 24	COMMON, ROUTINE, STANDARD: same, everyday, usual, average, ordinary, regular	Normal	Low
List 25	COMMENT, REMARK, RESPOND: answer, talk, say, speak, reply, tell	Talk	High
List 26	COMMENT, REMARK, SUGGEST: talk, answer, opinion, say, tell, speak	Talk	Low
List 27	CLARINET, TRUMPET, TUBA: instrument, music, horn, flute, band, blow	Music	High
List 28	TROMBONE, TRUMPET, TUBA: band, loud, flute, brass, clarinet, blow	Music	Low

The FAS values for each critical lure word (*lure word FAS* hereafter) were determined by the sum of the probabilities that each critical lure produced its six associated words in free association. Similarly, the FAS values for each list (*FAS list strength* hereafter) were calculated as the sum of the FAS values for the three critical lures (similar to Robinson and Roediger, 1997; Beato and Díez, 2011; Beato and Arndt, 2014). BAS values, measured as the probability that study items produced critical lures in free association, were similarly calculated for both critical lures and lists.

There were 14 “general themes” in the lists (e.g., *music*). For each theme, we built two different six-word study lists. One of the two study lists per general theme included six associates that had relatively stronger FAS relations to critical lures (*high-FAS lists* hereafter) and the other included six associates that had relatively weaker FAS relations to critical lures (*low-FAS lists* hereafter). For example, for the general theme “music” the high-FAS critical lures were CLARINET (FAS=0.660), TRUMPET (FAS=0.727), and TUBA (FAS=0.576), each of which had forward associations to the study items *instrument, music, horn, flute, band, and blow*. The low-FAS critical lures for the same theme were TROMBONE (FAS=0.275), TRUMPET (FAS=0.202), and TUBA (FAS=0.210), each of which had forward associations to the study items *band*, *loud*, *flute*, *brass*, *clarinet*, and *blow*. Lists constructed with high-FAS list strength (*M*=1.641; *SD*=0.367; ranged from 1.073 to 2.301), and low-FAS list strength (*M*=0.787; *SD*=0.183; ranged from 0.454 to 1.112) reliably differed from one another, *t*(26)=7.79; *p*<0.001, *d*=2.94, 95% CI [0.63, 1.08]. Further, there was a wide variability in FAS, such that FAS list strength ranged from 0.45 to 2.30^2^ and lure word FAS ranged from 0.127 to 0.865. Finally, we constructed lists in a way that controlled mean levels of BAS across the stimulus sets that varied in FAS (*M*=0.34 and *M*=0.29, for the high- and low-FAS lists, respectively), *t*(26)=0.37; *p*>0.05, although BAS still varied substantially across stimulus sets and for the critical lures within a stimulus set. Table 2 reports FAS and BAS values per critical lure.

**Table 2:** Forward associative strength (FAS) condition, mean percentages of true recognition (TR) and false recognition (FR) per list, mean percentages of false recognition per critical lure, and FAS and BAS values per critical lure were included.

List	FAS condition	TR list	FR list	FR Lure 1	FR Lure 2	FR Lure 3	FAS Lure 1	FAS Lure 2	FAS Lure 3	BAS Lure 1	BAS Lure 2	BAS Lure 3
List 1	High	78.33	5.00	15	0	0	0.626	0.865	0.810	0.015	0.000	0.107
List 2	Low	77.50	8.33	5	20	0	0.271	0.219	0.168	0.000	0.000	0.000
List 3	High	94.17	15.00	15	15	15	0.664	0.627	0.544	0.051	0.000	0.184
List 4	Low	75.00	21.67	10	50	5	0.337	0.288	0.354	0.032	0.686	0.000
List 5	High	75.00	16.67	30	10	10	0.544	0.421	0.684	0.014	0.000	0.054
List 6	Low	79.17	28.33	30	35	20	0.275	0.148	0.204	0.082	0.014	0.021
List 7	High	72.50	3.33	5	5	0	0.848	0.400	0.307	0.124	0.000	0.000
List 8	Low	73.33	10.00	15	10	5	0.368	0.446	0.229	0.116	0.000	0.000
List 9	High	81.67	38.33	20	40	55	0.658	0.355	0.516	0.067	0.056	0.196
List 10	Low	86.67	36.67	40	40	30	0.137	0.190	0.127	0.553	0.786	0.067
List 11	High	70.83	10.00	20	10	0	0.539	0.480	0.370	0.355	0.000	0.000
List 12	Low	71.67	8.33	10	10	5	0.393	0.210	0.322	0.000	0.162	0.000
List 13	High	70.00	21.67	25	25	15	0.753	0.246	0.391	0.024	0.015	0.000
List 14	Low	72.50	8.33	10	10	5	0.223	0.258	0.261	0.168	0.016	0.000
List 15	High	76.67	38.33	0	40	75	0.758	0.407	0.746	0.155	0.050	0.519
List 16	Low	64.17	25.00	15	15	45	0.194	0.676	0.242	0.000	0.155	0.092
List 17	High	81.67	45.00	45	20	70	0.265	0.476	0.739	0.090	0.000	0.988
List 18	Low	73.33	18.33	30	10	15	0.421	0.140	0.311	0.126	0.000	0.028
List 19	High	75.00	15.00	25	10	10	0.829	0.700	0.732	0.183	0.039	0.091
List 20	Low	83.33	28.33	40	20	25	0.375	0.236	0.220	0.170	0.047	0.100
List 21	High	64.17	25.00	5	15	55	0.755	0.294	0.341	0.015	0.000	0.356
List 22	Low	70.00	35.00	5	35	65	0.158	0.265	0.321	0.000	0.036	0.163
List 23	High	63.33	21.67	0	60	5	0.385	0.576	0.283	0.020	0.723	0.034
List 24	Low	69.17	10.00	15	10	5	0.212	0.254	0.145	0.220	0.092	0.000
List 25	High	55.83	10.00	5	0	25	0.276	0.247	0.550	0.000	0.000	0.000
List 26	Low	49.17	5.00	10	0	5	0.322	0.240	0.171	0.000	0.000	0.000
List 27	High	78.33	16.67	0	45	5	0.660	0.727	0.576	0.097	0.143	0.013
List 28	Low	75.00	16.67	20	30	0	0.275	0.202	0.210	0.014	0.165	0.000

Finally, we built five additional six-word lists, each with three critical lures (see Table 3), to be used as *unrelated distractors* and *unrelated critical-lure distractors* on the memory tests. Distractor lists were constructed to ensure that they were associatively unrelated to study items (Nelson et al., 1998) following a procedure similar to that used to construct study lists, such that FAS list strength ranged from 0.93 to 1.46. The recognition memory test included 168 words randomly intermixed: the 84 studied words, the 42 critical lures, and 42 distractors (15 unrelated critical-lure distractors, 27 unrelated distractors).

**Table 3:** Five six-word distractor lists with three critical lures, general theme, and FAS were included.

List	DISTRACTOR LIST CRITICAL LURES: Associated words	General theme	FAS distractor 1	FAS distractor 2	FAS distractor 3	FAS list distractors
List 1	ATTRACTIVE, GORGEOUS, SEXY: pretty, beautiful, girl, man, handsome, woman	Pretty	0.609	0.420	0.226	1.255
List 2	COUNTY, LOCAL, PROVIDENCE: city, state, place, country, area, town	Place	0.454	0.139	0.335	0.928
List 3	DRAWING, PAINTER, PAINTING: art, picture, artist, paint, color, canvas	Paint	0.497	0.422	0.538	1.457
List 4	CRUDE, REPULSIVE, VULGAR: rude, gross, disgusting, mean, ugly, nasty	Disgusting	0.510	0.546	0.393	1.449
List 5	AX, CHISEL, HATCHET: hammer, knife, chop, tool, cut, saw	Tools	0.263	0.466	0.240	0.969

### Procedure

First, participants were informed about the nature and procedure of the study and signed a consent form. Participants were tested individually and were instructed that their task was to remember the words as best they could, because they would be given a memory test later in the experiment.

Each participant was presented with the study items from 14 lists, one list per general theme (seven high-FAS lists and seven low-FAS lists). General themes’ high-FAS lists and low-FAS lists were presented equally often across participants. Further, we confirmed that no associates or critical lures were repeated within the stimuli experienced by a participant. Study items were presented individually on a computer screen for 2,000ms with a 500-ms ISI blocked by DRM list. The associates within each list were arranged in decreasing order of FAS. The order of list presentation was randomized for each participant. At the conclusion of the study phase, participants completed a self-paced recognition memory test, where they were asked to determine whether each word was previously studied by pressing the “O” key to indicate it was OLD, and the “N” key to indicate it was NEW.

### Power Analysis

We evaluated the power to detect the effects of FAS on false recognition using the three strategies highlighted above using G*Power (Faul et al., 2007, 2009). When participants were used as the unit of analysis, our sample size of 40 was sufficient to detect a large effect (*d_z_*=0.5), with power=0.869. However, the power to detect a medium-sized effect (*d_z_*=0.3) was considerably smaller, 0.457. When study lists (*N*=28) were used as the unit of analysis for correlations, the power to detect a large effect (*ρ*=0.5) was 0.799, which is near the conventionally-preferred level of 0.80 (Cohen, 1988). However, the power to detect a medium-sized effect (*ρ*=0.3) was considerably lower, 0.348. Finally, when critical lures (*N*=84) were used as the unit of analysis for correlations, the power to detect a large effect (*ρ*=0.5) and a medium-sized effect (*ρ*=0.3) were both sufficient by conventional standards, with power of 0.999 and 0.800, respectively.

### Data Analysis

Given the relatively modest levels of power to detect medium-sized effects in most of our analyses, we chose to analyze our data using both standard null-hypothesis tests and Bayesian analysis (Kass and Raftery, 1995), which allows quantification of the strength of the evidence for the null and alternative hypothesis. We conducted standard analyses that treat FAS as a categorical variable (e.g., *t*-tests) and as a continuous variable (correlation) to assess its relationship to false recognition. We also conducted correlational analyses on a variety of other characteristics of our stimuli (Nelson et al., 1998) to evaluate how well semantic memory variables other than FAS predicted false recognition (see, e.g., Brainerd et al., 2008). As highlighted above, these analyses were conducted using study lists’ characteristics as the unit of analysis and using critical lures’ characteristics as the unit of analysis. Finally, we conducted Bayesian analyses using JASP (Version 0.14.1; JASP Team, 2020) to quantify the strength of the evidence for the observed statistical outcomes using *BF*_10_. *BF*_10_>1 supports the alternate hypothesis, and a *BF*_10_<1 supports the null hypothesis. Importantly, *BF*_10_ between 3 and 20 is signifies positive evidence for the alternate hypothesis, *BF*_10_ between 20 and 150 signifies strong evidence for the alternate hypothesis, and *BF*_10_ greater than 150 signifies very strong evidence for the alternate hypothesis (Kass and Raftery, 1995). Similarly, *BF*_10_ between 0.33 and 0.05 is signifies positive evidence for the null hypothesis, *BF*_10_ between 0.05 and 0.0067 signifies strong evidence for the null hypothesis, and *BF*_10_ below 0.0067 signifies very strong evidence for the null hypothesis.^3^

## Results

Table 2 reports the mean percentage of true recognition per list and false recognition per critical lure and list, while Table 4 reports the mean percentage of true and false recognition as a function of FAS and whether items were studied, critical lures, unrelated critical-lure distractors, or unrelated distractors.

**Table 4:** Mean percentage of true recognition and false recognition as a function of FAS, as well as baseline false alarm rates to unrelated critical-lure distractors and unrelated distractors.

	True recognition	False recognition
High FAS	74.11 (9.36)	20.12 (12.72)
Low FAS	72.86 (8.91)	18.57 (10.70)
Unrelated critical-lure distractors		5.67 (7.78)
Unrelated distractors		5.92 (7.45)

Standard deviations are reported in parenthesis.

### False Memory Effect

A one-way repeated-measures analysis of variance (ANOVA) was used to compare the percentage of old judgments to studied words, critical lures, unrelated critical-lure distractors, and unrelated distractors. This analysis revealed a significant difference, *F*(3, 117)=521.535; *p*<0.001, *η*^2^*_p_*=0.930. Bonferroni *post-hoc* tests showed that hits to studied words (true recognition; *M*=73.48, *SD*=13.06) were higher than false alarms to critical lures (false recognition; *M*=19.35, *SD*=10.64), unrelated critical-lure distractors (*M*=5.67, *SD*=7.78) and unrelated distractors (*M*=5.92, *SD*=7.45; *p*<0.001 for all comparisons). There were also significant differences between false alarms to critical lures and both unrelated critical-lure distractor and unrelated distractor items (*p*<0.001). There was not a reliable difference between the two types of unrelated distractors (*p*>0.05). Thus, the stimuli we constructed for this study produced the typical DRM false memory effect.

### True Recognition, False Recognition, and FAS

The percentage of hits (true recognition) and false alarms to critical lures (false recognition) as a function of FAS is presented in Table 4. FAS did not impact hits, *t*(39)=0.630; *p*=0.532, *d*=0.09, *BF*_10_=0.206, or false alarms to critical lures, *t*(39)=0.868; *p*=0.391, *d*=0.13, *BF*_10_=0.242. The *BF*_10_ values for hits and false alarms to critical lures indicate positive support for the conclusion that FAS failed to impact true and false recognition.

We also examined the relationship between FAS and false recognition using correlation. Our DRM lists included three critical lures per list, which allowed us to correlate FAS list strength and lure word FAS with false recognition separately. Neither of these analyses produced a significant correlation [*r*(26)=−0.026, *p*=0.895, *BF*_10_=0.237 for FAS list strength; *r*(82)=0.047, *p*=0.668, and *BF*_10_=0.149 for lure word FAS]. The *BF*_10_ values for these correlations again indicate positive evidence that FAS was unrelated to false recognition. It is important to note that these null correlations occurred despite there being substantial variability in false recognition across lists and critical lures. For example, some high-FAS lists yielded high levels of false recognition, such as the list with the critical lures BURGLAR, THEFT, and THIEF (45%), whereas other high-FAS lists produced very low levels of false recognition (e.g., SOCCER, SOFTBALL, and VOLLEYBALL list, 3%). In low-FAS lists, we also found wide differences in false recognition, ranging between 37% (DEAD, DEATH, and DIE list) and 5% (COMMENT, REMARK, and SUGGEST list).

Although FAS was unrelated to false recognition, we sought to explore whether other stimulus characteristics were related to false recognition. Thus, we correlated the characteristics of the study words included in the lists with the overall level of false recognition produced by that list (i.e., averaged across the three critical lures). The variables examined in these analyses were BAS, interconnectivity among the associates included in the lists (sum of the FAS and BAS of all possible pairings of study items and critical lures), associates’ set size, associates’ concreteness, the mean connectivity among lists’ associates, the probability of a resonant connection, and resonant connection strength (Nelson et al., 1998). The only reliable correlation found in these analyses was between false recognition and BAS, *r*(26)=0.643, *p*<0.001, *BF*_10_=152.01, indicating very strong evidence that false recognition and BAS were related.^4^

We also conducted correlational analyses between false recognition and critical lure characteristics. We computed each critical lure’s average BAS with study items, frequency of occurrence, concreteness, set size (i.e., number of different words produced by a critical lure), density (i.e., mean connectivity among all critical lure associates), accessibility index (i.e., number of word that produced the critical lure as a response), resonant connections (i.e., number of critical lure’s associates that produced it as an associate), and resonant strength (i.e., associative strength from all the words produced by the critical lure to the critical lure; Nelson et al., 1998). This analysis showed there were significant correlations between false recognition and critical lures’ BAS, *r*(82)=0.662, *p*<0.001, BF_10_>1,422,000,000, frequency, *r*(82)=0.388, *p*<0.001, BF_10_=95.27, resonant connections, *r*(82)=0.463, *p*<0.001 BF_10_=2099.64, resonant strength, *r*(82)=0.470, *p*<0.001, BF_10_=2982.68, and accessibility, *r*(82)=0.479, *p*<0.001, BF_10_=4735.24. For each correlation, BF_10_ indicated strong or very strong evidence each variable was positively related to false recognition.

## Discussion

The empirical and theoretical aim of this research was to analyze the effect of FAS on false recognition. In order to do this, we constructed stimulus sets that varied widely in FAS. The results of this study showed that false recognition was robust. Moreover, there was wide variability in false recognition rates per list, ranging from 3 to 45%. Thus, there was substantial variability in both false recognition and FAS, which is critical for assessing if there is a relationship between FAS and false recognition.

Despite empirical conditions that were conducive to observing a relationship between FAS and false recognition, no such relationship was found. This finding replicates previous research that has failed to find a relationship between FAS and false recognition (e.g., Roediger et al., 2001; Gallo and Roediger, 2002; Beato and Arndt, 2014), but stands in contrast to research that has found a reliable relationship between FAS and false recognition (e.g., Brainerd and Wright, 2005; Arndt, 2012b, 2015). Importantly, interpretation of the present results is not complicated by restricted range in FAS, a concern that has been advanced to explain the finding of Roediger et al. (2001) that FAS was not correlated with false recognition (see Brainerd and Wright, 2005). Finally, the present results extend prior findings of a null correlation between FAS and false recognition to DRM lists related to multiple critical lures.

Although FAS failed to predict false memory, our correlational analyses produced several notable results. Most importantly, BAS *was* associated with false recognition. This association is particularly notable because we sought to control the mean levels of this variable across the high- and low-FAS conditions. Despite this constraint, BAS was strongly correlated with false recognition, replicating extensive evidence that BAS is a reliable predictor of false memory (e.g., Roediger et al., 2001; Gallo and Roediger, 2002; Arndt, 2012b, 2015; Beato and Arndt, 2017). Beyond BAS, our correlational analyses found that the factors that were correlated with greater false recognition generally measured the extent to which a critical lure is activated by the study of its associates, such as resonant connections and resonant strength. Thus, these measures may reflect, like BAS, how active a critical lure’s representation is following study of its associates (Roediger et al., 2001).

At a theoretical level, the present results fit most naturally with associative activation views of false memory (Roediger et al., 2001; Howe et al., 2009b). These views posit that spreading activation from study item representations to critical lure representations plays a key role in producing false memory. Two cardinal predictions from these theories are upheld by the present data. First, that the primary driver of false memory is the extent to which study items activate lure representations in semantic memory, and thus the extent to which lure items can be confused with episodically-experienced items. This activation is most directly measured by BAS in word association norms. Second, that associative variables, which are unrelated to how much study items activate critical lures’ representations, such as FAS, will not affect false memory. Both of these predictions were supported in the present study, despite the fact that we implemented a strong manipulation of FAS between lists and sought to control BAS across levels of that manipulation.

In addition to favoring associative-activation theories of false memory, the present results are puzzling from the perspective of theories highlighting the role that the similarity between study items and critical lures in semantic memory plays in producing false memory (Arndt and Hirshman, 1998; Brainerd et al., 2008; Arndt, 2012a; Brainerd et al., 2020). In particular, these views suggest that false memory increases with the similarity between study items and lure items, as well as the extent to which study lists’ gist is encoded during study (Brainerd et al., 2020). Thus, FAS, BAS, and other measures of semantic memory activation should increase critical lure false memory. In contrast to this expectation, FAS failed to produce differences in false memory in this study, despite our intentional and substantial manipulation of this variable. In addition, FAS failed to correlate with false recognition, both when measured based upon study list characteristics and when measured based upon critical lure characteristics. Finally, in our analysis of list-wide semantic memory variables with false recognition as well as critical lures’ semantic memory characteristics, the only correlation we found in *both* sets of analyses was between BAS and false recognition.

One set of outcomes from the present study may be taken as partial evidence favoring the view that similarity among study items enhances gist encoding, which is hypothesized to play a role in false memory production (Brainerd et al., 2008, 2020). In our analyses of semantic memory variables associated with false memory, several semantic memory variables, such as critical lures’ word frequency, resonant connections, resonant strength, and accessibility were all correlated with false recognition. While this broader set of semantic memory variables associating with false recognition is consistent with general semantic memory activation underlying false recognition (Brainerd et al., 2008), it is critically important that other key semantic variables, such as connectivity, failed to correlate with false recognition (Brainerd et al., 2020).^5^ Indeed, it has been suggested that connectivity can serve as a proxy measure for a study lists’ gist, since it assesses inter-relationships among studied items, which can be viewed as assessing, in part, the semantic relationships among studied items that are thought to underlie a study list’s overall gist (Brainerd et al., 2020). Thus, while views proposing that non-associative semantic memory activation underlies false memory are consistent with some aspects of the present data, the correlations observed in our results were (1) not as wide-ranging as would be expected if semantic memory activation is the primary basis for false memory and (2) not reliable for key variables thought to be proxy-measures of gist processing during encoding.

In closing, we wish to emphasize four key points. First, we failed to observe a correlation between FAS and false recognition, despite using conditions that provide an excellent opportunity for such a relationship to be found. Second, we observed a positive relationship between BAS and false recognition, despite not directly attempting to manipulate BAS in this study. Third, both of these results occurred when we assessed the relationship between list-wide associative strength and false recognition as well as when we assessed the relationship at the level of individual critical lures. Importantly, because the FAS and BAS results occurred regardless of the method we used to calculate FAS, BAS, and false recognition, it suggests that the relationships we observed in this study are products of the nature of the associations between study lists and critical lures. Fourth, and finally, these results favor activation-based explanations of false memory (Roediger et al., 2001; Howe et al., 2009b) over similarity-based explanations (Arndt and Hirshman, 1998; Brainerd et al., 2008; Arndt, 2012a; Brainerd et al., 2020). Thus, these results best support the view that study items in the DRM paradigm activate critical lures’ representations during encoding, which leads critical lures to be falsely recognized on a subsequent memory test.

## Data Availability Statement

The data collected for this study will be made available by the authors on request.

## Ethics Statement

The research reported in this paper was reviewed and approved by the Middlebury College Institutional Review Board. The participants provided their written informed consent to participate in this study.

## Author Contributions

The authors contributed equally to this work.

## Funding

This research was partially supported by the University of Salamanca and by grant SA052G18, awarded to MSB by Junta de Castilla y León.

## Conflict of Interest

The authors declare that the research was conducted in the absence of any commercial or financial relationships that could be construed as a potential conflict of interest.

## Publisher’s Note

All claims expressed in this article are solely those of the authors and do not necessarily represent those of their affiliated organizations, or those of the publisher, the editors and the reviewers. Any product that may be evaluated in this article, or claim that may be made by its manufacturer, is not guaranteed or endorsed by the publisher.

## Footnotes

^1^The astute reader will notice that some words are repeated across the two sets of critical lures and study items. As is clarified in the method section, participants studied either the strong or weak FAS lists for a given general theme. Thus, the overlap in specific stimulus items is not problematic for assessing the effects of FAS on false recognition.

^2^The range of mean FAS values was between 0.025 and 0.128. The use of the sum or mean of FAS values to determine FAS list strength and lure word FAS does not alter in any way the conclusions obtained in the different analyzes as they are linear transformations of one another.

^3^The *BF*_10_ values that quantify support for the null hypothesis are computed as the multiplicative inverse of the *BF*_10_ values that quantify support for the alternate hypothesis.

^4^While it may seem unusual that BAS was related to false recognition because we controlled BAS across the high- and low-FAS conditions, the method we used to equate BAS across FAS conditions only ensured that the *mean* BAS values were comparable across high- and low-FAS lists. Thus, this method left substantial variability in BAS across lists, which allowed the possibility that correlational analyses would reveal the impact of BAS on false recognition.

^5^Connectivity was not correlated with false recognition when study lists were used as the unit of analysis [*r*(26) = 0.025, *p* = 0.901, *BF*_10_ = 0.236] and was negatively correlated with false recognition when critical lures were used as the unit of analysis, albeit not significantly so [*r*(82) = −0.180, *p* = 0.102, *BF*_10_ = 0.507]. While *BF*_10_ for this latter correlation falls in the range, where it fails to provide support for the null hypothesis, evaluating the statistical hypothesis that connectivity and false recognition were positively correlated, as gist-based perspectives predict, produces a *value of p* of 0.949 and a *BF*_10_ = 0.054, which falls in the range of positive evidence that the two variables are unrelated, and is close to the range where the evidence is considered “strong.”

## References

[ref1] Arndt J. (2006). Distinctive information and false recognition: the role of encoding and retrieval processes. J. Mem. Lang. 54, 113–130. doi: 10.1016/j.jml.2005.08.003

[ref2] Arndt J. (2012a). “False recollection: empirical findings and their theoretical implications” in Psychology of Learning and Motivation. ed. Ross B. H. *Vol*. 56 (Amsterdam, The Netherlands: Academic Press), 81–124.

[ref3] Arndt J. (2012b). The influence of forward and backward associative strength on false recognition. J. Exp. Psychol. Learn. Mem. Cogn. 28, 747–756. doi: 10.1037/a0026375, PMID: 22103785 PMC3383060

[ref4] Arndt J. (2015). The influence of forward and backward associative strength on false memories for encoding context. Memory 23, 1093–1111. doi: 10.1080/09658211.2014.959527, PMID: 25312499 PMC4983188

[ref5] Arndt J. Beato M. S. (2017). The role of language proficiency in producing false memories. J. Mem. Lang. 95, 146–158. doi: 10.1016/j.jml.2017.03.004

[ref6] Arndt J. Gould C. (2006). An examination of two process theories of false recognition. Memory 14, 814–833. doi: 10.1080/09658210600680749, PMID: 16938694

[ref7] Arndt J. Hirshman E. (1998). True and false recognition in MINERVA2: explanations from a global-matching perspective. J. Mem. Lang. 39, 371–391. doi: 10.1006/jmla.1998.2581

[ref8] Beato M. S. Arndt J. (2014). False recognition production indexes in forward associative strength (FAS) lists with three critical words. Psicothema 26, 457–463. doi: 10.7334/psicothema2014.79, PMID: 25340891

[ref9] Beato M. S. Arndt J. (2017). The role of backward associative strength in false recognition of DRM lists with multiple critical lures. Psicothema 29, 358–363. doi: 10.7334/psicothema2016.248, PMID: 28693707

[ref10] Beato M. S. Arndt J. (2021). The effect of language proficiency and associative strength on false memory. Psychol. Res. doi: 10.1007/s00426-020-01449-3 [Epub ahead of print], PMID: 33387022

[ref11] Beato M. S. Díez E. (2011). False recognition production indexes in Spanish for 60 DRM lists with three critical words. Behav. Res. Methods 43, 499–507. doi: 10.3758/s13428-010-0045-9, PMID: 21298572

[ref12] Beato M. S. Boldini A. Cadavid S. (2012). False memory and level of processing effect: an event-related potential study. Neuroreport 23, 804–808. doi: 10.1097/WNR.0b013e32835734de, PMID: 22811058

[ref13] Brainerd C. J. Wright R. (2005). Forward association, backward association, and the false-memory illusion. J. Exp. Psychol. Learn. Mem. Cogn. 31, 554–567. doi: 10.1037/0278-7393.31.3.554, PMID: 15910137

[ref14] Brainerd C. J. Yang Y. Reyna V. F. Howe M. L. Mills B. A. (2008). Semantic processing in “associative” false memory. Psychon. Bull. Rev. 15, 1035–1053. doi: 10.3758/PBR.15.6.1035, PMID: 19001566

[ref15] Brainerd C. J. Chang M. Bialer D. M. (2020). From association to gist. J. Exp. Psychol. Learn. Mem. Cogn. 46, 2106–2127. doi: 10.1037/xlm0000938, PMID: 32658546

[ref16] Cadavid S. Beato M. S. (2017). False recognition in DRM lists with low association: A normative study. Psicológica 38, 133–147.

[ref17] Cadavid S. Beato M. S. Fernández A. (2012). Falso reconocimiento en listas DRM con tres palabras críticas: Asociación directa vs. inversa [false recognition in DRM lists with three critical words: forward vs. backward association]. Psicológica 33, 39–58.

[ref18] Coane J. H. McBride D. M. Huff M. J. Chang K. Marsh E. M. Smith K. A. (2021). Manipulations of list type in the DRM paradigm: A review of how structural and conceptual similarity affect false memory. Front. Psychol. 12:668550. doi: 10.3389/fpsyg.2021.668550, PMID: 34135826 PMC8200635

[ref19] Cohen J. (1988). Statistical Power Analysis for the Behavioral Sciences (2nd *Edn*). Hillsdale, NJ: Lawrence Erlbaum Associates.

[ref20] Deese J. (1959). On the prediction of occurrence of particular verbal intrusions in immediate recall. J. Exp. Psychol. 58, 17–22. doi: 10.1037/h0046671, PMID: 13664879

[ref21] Faul F. Erdfelder E. Lang A.-G. Buchner A. (2007). G*Power 3: A flexible statistical power analysis program for the social, behavioral, and biomedical sciences. Behav. Res. Methods 39, 175–191. doi: 10.3758/BF03193146, PMID: 17695343

[ref22] Faul F. Erdfelder E. Buchner A. Lang A.-G. (2009). Statistical power analyses using G*Power 3.1: tests for correlation and regression analyses. Behav. Res. Methods 41, 1149–1160. doi: 10.3758/BRM.41.4.1149, PMID: 19897823

[ref23] Gallo D. A. Roediger H. L. III (2002). Variability among word lists in eliciting memory illusions: evidence for associative activation and monitoring. J. Mem. Lang. 47, 469–497. doi: 10.1016/S0749-596X(02)00013-X

[ref24] Howe M. L. Wimmer M. C. Blease K. (2009a). The role of associative strength in children’s false memory illusions. Memory 17, 8–16. doi: 10.1080/09658210802438474, PMID: 19031309

[ref25] Howe M. L. Wimmer M. C. Gagnon N. Plumpton S. (2009b). An associative-activation theory of children’s and adults’ memory illusions. J. Mem. Lang. 60, 229–251. doi: 10.1016/j.jml.2008.10.002

[ref26] Huff M. J. Bodner G. E. Gretz M. R. (2020). Reducing false recognition in the Deese-Roediger/McDermott paradigm: related lures reveal how distinctive encoding improves encoding and monitoring processes. Front. Psychol. 11, 602347. doi: 10.3389/fpsyg.2020.602347, PMID: 33329270 PMC7714777

[ref27] JASP Team (2020). JASP (Version 0.14.1) [Computer software].

[ref28] Kass R. E. Raftery A. E. (1995). Bayes factors. J. Am. Stat. Assoc. 90, 773–795. doi: 10.1080/01621459.1995.10476572

[ref29] Knott L. M. Dewhurst S. A. Howe M. L. (2012). What factors underlie associative and categorical memory illusions? The roles of backward associative strength and inter-item connectivity. J. Exp. Psychol. Learn. Mem. Cogn. 38, 229–239. doi: 10.1037/a0025201, PMID: 21875250

[ref30] McEvoy C. L. Nelson D. L. Komatsu T. (1999). What is the connection between true and false memories? The differential roles of interitem associations in recall and recognition. J. Exp. Psychol. Learn. Mem. Cogn. 25, 1177–1194. doi: 10.1037//0278-7393.25.5.1177, PMID: 10505342

[ref31] Nelson D. L. McEvoy C. L. Schreiber T. A. (1998). The University of South Florida word association, rhyme, and word fragment norms. Available at: http://w3.usf.edu/FreeAssociation (Accessed October 19, 2005). 10.3758/bf03195588 15641430

[ref32] Pitarque A. Satorres E. Sales A. Escudero J. Meléndez J. C. (2018). Effects of stimuli repetition and age in false recognition. Psychol. Rep. 121, 1106–1119. doi: 10.1177/0033294117747284, PMID: 29298586

[ref33] Robinson K. J. Roediger H. L. III (1997). Associative processes in false recall and false recognition. Psychol. Sci. 8, 231–237. doi: 10.1111/j.1467-9280.1997.tb00417.x

[ref34] Roediger H. L. III McDermott K. B. (1995). Creating false memories: remembering words not presented in lists. J. Exp. Psychol. Learn. Mem. Cogn. 21, 803–814. doi: 10.1037/0278-7393.21.4.803

[ref35] Roediger H. L. III Watson J. M. McDermott K. B. Gallo D. A. (2001). Factors that determine false recall: a multiple regression analysis. Psychon. Bull. Rev. 8, 385–407. doi: 10.3758/BF03196177, PMID: 11700893

